# Preliminary Screening for Hereditary Breast and Ovarian Cancer Using a Chatbot Augmented Intelligence Genetic Counselor: Development and Feasibility Study

**DOI:** 10.2196/25184

**Published:** 2021-02-05

**Authors:** Ann Sato, Eri Haneda, Nobuyasu Suganuma, Hiroto Narimatsu

**Affiliations:** 1 Department of Genetic Medicine Kanagawa Cancer Center Yokohama, Kanagawa Japan; 2 Department of Breast and Endocrine Surgery Kanagawa Cancer Center Yokohama, Kanagawa Japan; 3 Cancer Prevention and Cancer Control Division Kanagawa Cancer Center Research Institute Yokohama, Kanagawa Japan; 4 Graduate School of Health Innovation Kanagawa University of Human Services Kawasaki, Kanagawa Japan

**Keywords:** artificial intelligence, augmented intelligence, hereditary cancer, familial cancer, IBM Watson, preliminary screening, cancer, genetics, chatbot, screening, feasibility

## Abstract

**Background:**

Breast cancer is the most common form of cancer in Japan; genetic background and hereditary breast and ovarian cancer (HBOC) are implicated. The key to HBOC diagnosis involves screening to identify high-risk individuals. However, genetic medicine is still developing; thus, many patients who may potentially benefit from genetic medicine have not yet been identified.

**Objective:**

This study’s objective is to develop a chatbot system that uses augmented intelligence for HBOC screening to determine whether patients meet the National Comprehensive Cancer Network (NCCN) BRCA1/2 testing criteria.

**Methods:**

The system was evaluated by a doctor specializing in genetic medicine and certified genetic counselors. We prepared 3 scenarios and created a conversation with the chatbot to reflect each one. Then we evaluated chatbot feasibility, the required time, the medical accuracy of conversations and family history, and the final result.

**Results:**

The times required for the conversation were 7 minutes for scenario 1, 15 minutes for scenario 2, and 16 minutes for scenario 3. Scenarios 1 and 2 met the BRCA1/2 testing criteria, but scenario 3 did not, and this result was consistent with the findings of 3 experts who retrospectively reviewed conversations with the chatbot according to the 3 scenarios. A family history comparison ascertained by the chatbot with the actual scenarios revealed that each result was consistent with each scenario. From a genetic medicine perspective, no errors were noted by the 3 experts.

**Conclusions:**

This study demonstrated that chatbot systems could be applied to preliminary genetic medicine screening for HBOC.

## Introduction

Breast cancer is the most common form of cancer in Japan, with approximately 90,000 new cases every year. Approximately 10,000 patients in Japan die from breast cancer each year [1]. Approximately 5%-10% of breast cancers are strongly related to genetic background, and of these, hereditary breast and ovarian cancer (HBOC) is the most common [2-4]. HBOC is an autosomal-dominant disease that is diagnosed based on the presence of BRCA1 or BRCA2 (BRCA1/2) pathogenic germline mutations. The BRCA1/2 genes encode 2 proteins involved in DNA damage repair. Recent studies have shown that mutations in BRCA1/2 and several other genes, including TP53 and PALB2, can lead to hereditary breast cancer in Japanese women [5]. Reportedly, BRCA1 and BRCA2 mutation carriers have cumulative breast cancer risks of 72% and 69%, respectively, and cumulative ovarian cancer risks of 44% and 17%, respectively, up to the age of 80 years. These risks are remarkably higher than those in the general population [6]. Therefore, genetic counseling and testing are recommended for individuals with suspected HBOC and relatives of carriers of the BRCA1/2 mutation. For carriers of the BRCA1/2 mutation diagnosed by genetic testing, appropriate countermeasures such as surveillance or risk reduction surgery can be considered [7].

A diagnosis of HBOC relies on a screening procedure to identify those at a high risk of disease, and therefore, the collection of information about family history is very important. However, genetic medicine is still developing in Japan. Even in the United States, many patients who might potentially benefit from genetic medicine have not been identified. A previous study suggested that this gap may be caused by a lack of awareness and knowledge among health care workers and patients, delays in updating information due to frequent revisions of the BRCA1/2 testing criteria, and a lack of human resources and health care workers responsible for identifying these patients [8].

In Japan, the preliminary screening of patients at high risk of HBOC is usually conducted by a certified genetic counselor (CGC). High-risk patients who undergo screening are recommended to receive genetic counseling to enable them to decide whether to undergo genetic testing. As of April 2020, however, there were only 267 CGCs in Japan [9], and it is difficult to rapidly increase this number because advanced specialized education is provided at a limited number of facilities.

In recent years, we have focused on developing augmented intelligence, which has been applied for practical use. Augmented intelligence has several functions, including a chatbot mechanism, which is software that allows users to interact with the system through an algorithm without the need for human back-end intervention. Chatbots have been applied in previous research as a communication support tool for patients with cancer [10-12]. Reportedly, chatbots improved medication adherence in patients with breast cancer and were able to provide support and anxiety reduction in young adults who underwent cancer treatment. The chatbot was useful for both younger patients with cancer as well as for the health monitoring of older patients (≥ 65 years) with cancer. These studies suggested that chatbots are useful in the medical field, especially in supporting patients with cancer.

There are many advantages of using chatbots for preliminary screening in medical practices. First, the chatbot can independently conduct preliminary screening automatically and could thus handle some of the CGC’s routine work. The CGC would then be able to focus on more intricate work, such as personalized genetic counseling, which would improve productivity. Second, a chatbot for preliminary screening is easy to create because the purpose of the conversation is clear, and the flow of information collection used for determining whether the BRCA1/2 testing criteria are met has a pattern. Lastly, the chatbot will enable the identification of a larger number of patients; traditional preliminary screening requires time and effort, which could be resolved with the chatbot.

This study aims to develop a system based on IBM Watson (the chatbot system developed by IBM Corp) that would inquire about patients’ family and medical histories and would identify which patients with breast cancer should be contacted by a CGC. The friendly interface created using augmented intelligence would be easy for the patients to understand. The study endpoint involves an evaluation of the developed system's clinical feasibility, which would ideally increase the number of targeted people who are identified by the preliminary screening and thus enable the provision of genetic medicine to a larger number of people in the future.

## Methods

### Chatbot System

This is a feasibility study by simulation using a scenario. The study’s endpoint is to assess the accuracy of the family history heard by the chatbot system. Furthermore, as a preclinical study, we listened to the medical and family history of the actual medical staff and evaluated its accuracy.

We developed a system using the chatbot function of Watson, a service of IBM Corp’s cognitive computing system. In this study, we used the real-time conversational interface LINE, a social network service (SNS) provided by LINE Corporation. LINE is the most popular SNS in Japan, and we assumed that it would be familiar to the patients [13]. Using this interface, we established a system that targeted patients with breast cancer who visited a designated hospital and assessed whether they met the National Comprehensive Cancer Network (NCCN) guidelines for BRCA1/2 testing criteria, version 3.2019, using information given in the patients’ replies. The patients’ responses were stored in the terminal (ie, a smartphone or tablet). Finally, the medical history, the family history, and the final results of preliminary screening appeared on the terminal screen. The development of this system was supported by the Advanced Integration Technology (AIT) Corporation. We set the genetic counselor, named “AI,” as the persona to ensure that the patients could type to and interact with a familiar entity. Textbox 1 shows the persona of AI.

This study was approved by the institutional review board for the research of Kanagawa Cancer Center (2018 - epidemiology 55).

**Summary of the characteristics of the chatbot genetic counselor, AI.**

**Name**: AI

**Age**: 26 years old

**Gender**: Female

**Education**: Graduate school

**Occupation**: Genetic counselor working in a general hospital

**Hometown**: Elsewhere in Kanagawa Prefecture, Japan

**Family structure**: Father (56 years old), Mother (58 years old, nurse), older sister (29 years old, has 1 daughter), younger brother (18 years old, college student), Grandfather (died when AI was a high school student), Grandmother

**Lifestyle**: Lives with brother

**Hobby**: Cooking

### Modification of the Guideline

In this study, we partially modified the BRCA1/2 testing criteria according to our clinical practice at Kanagawa Cancer Center. The following items were omitted because the chatbot was not able to ask these questions of the patients: (1) individual from a family with a known BRCA1/2 pathogenic/likely pathogenic variant, including such variants found on research testing; (2) personal history of breast cancer diagnosed at 41-50 years old with ≥1 close blood relative with high-grade (Gleason score ≥7) prostate cancer; (3) personal history of breast cancer diagnosed at ≤60 years old with triple-negative breast cancer; (4) personal history of breast cancer diagnosed at any age with ≥1 close blood relative with metastatic prostate cancer or ≥2 additional diagnoses of breast cancer at any age in close blood relatives; (5) personal history of metastatic prostate cancer; (6) personal history of high-grade prostate cancer (Gleason score ≥7) at any age with ≥1 close blood relative with ovarian carcinoma, pancreatic cancer, or metastatic prostate cancer at any age or breast cancer <50 years old, or ≥2 close blood relatives with breast or prostate cancer (any grade) at any age; (7) BRCA1/2 pathogenic/likely pathogenic variant detected by tumor profiling on any tumor type in the absence of germline pathogenic/likely pathogenic variant analysis; (8) regardless of family history, some individuals with BRCA-related cancer who may benefit from genetic testing to determine targeted treatment eligibility.

The following item was omitted because it is rarely encountered in clinical practice in Japan: (1) personal history of breast cancer with Ashkenazi Jewish ancestry; (2) personal history of high-grade prostate cancer (Gleason score ≥7) at any age with Ashkenazi Jewish ancestry.

The final results were presented to demonstrate whether the patient met these modified criteria.

### Evaluation of Feasibility

**Figure 1:**
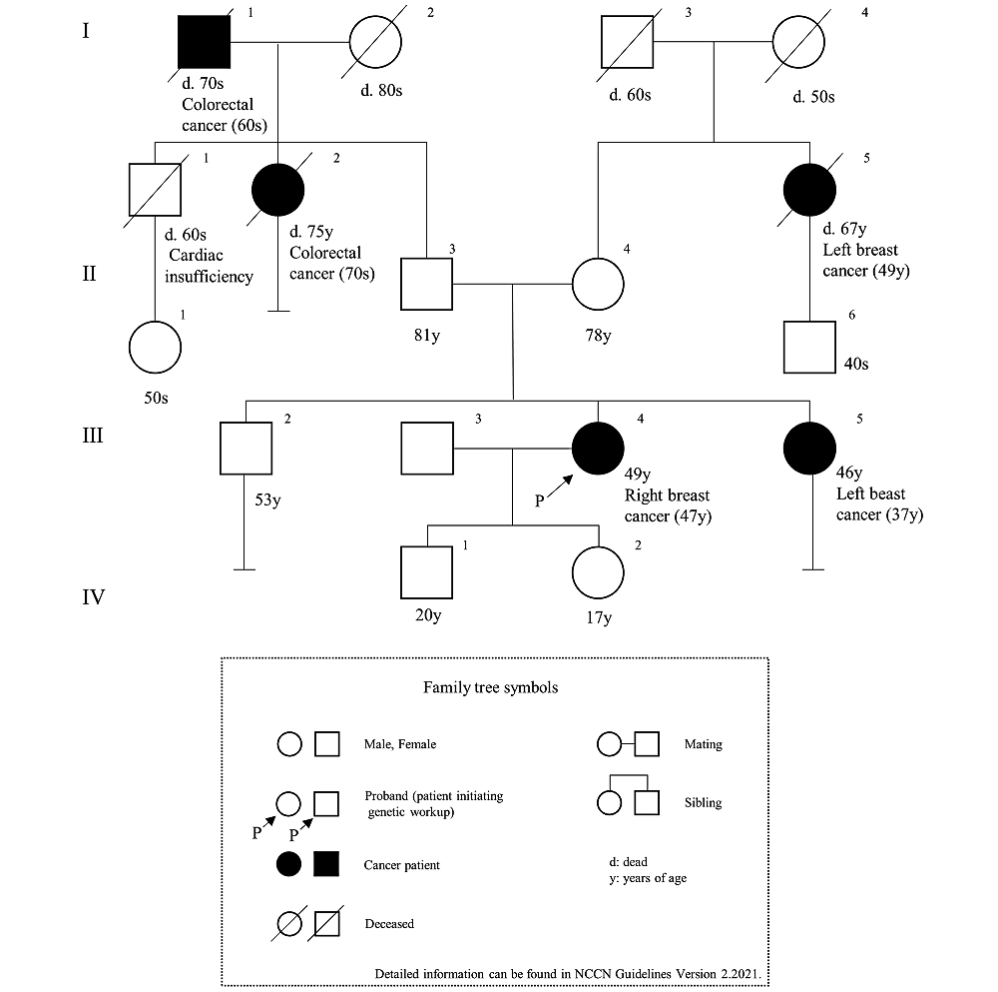
The family tree in scenario 1.

**Figure 2:**
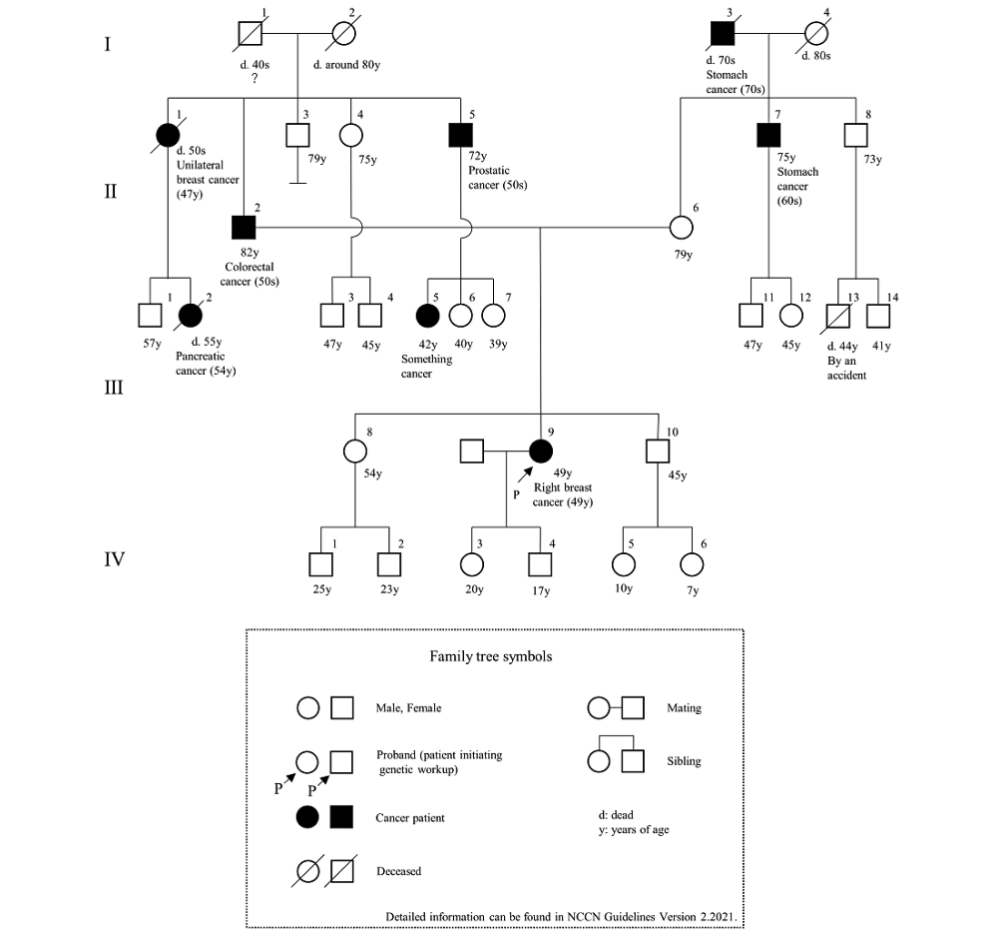
The family tree in scenario 2.

**Figure 3:**
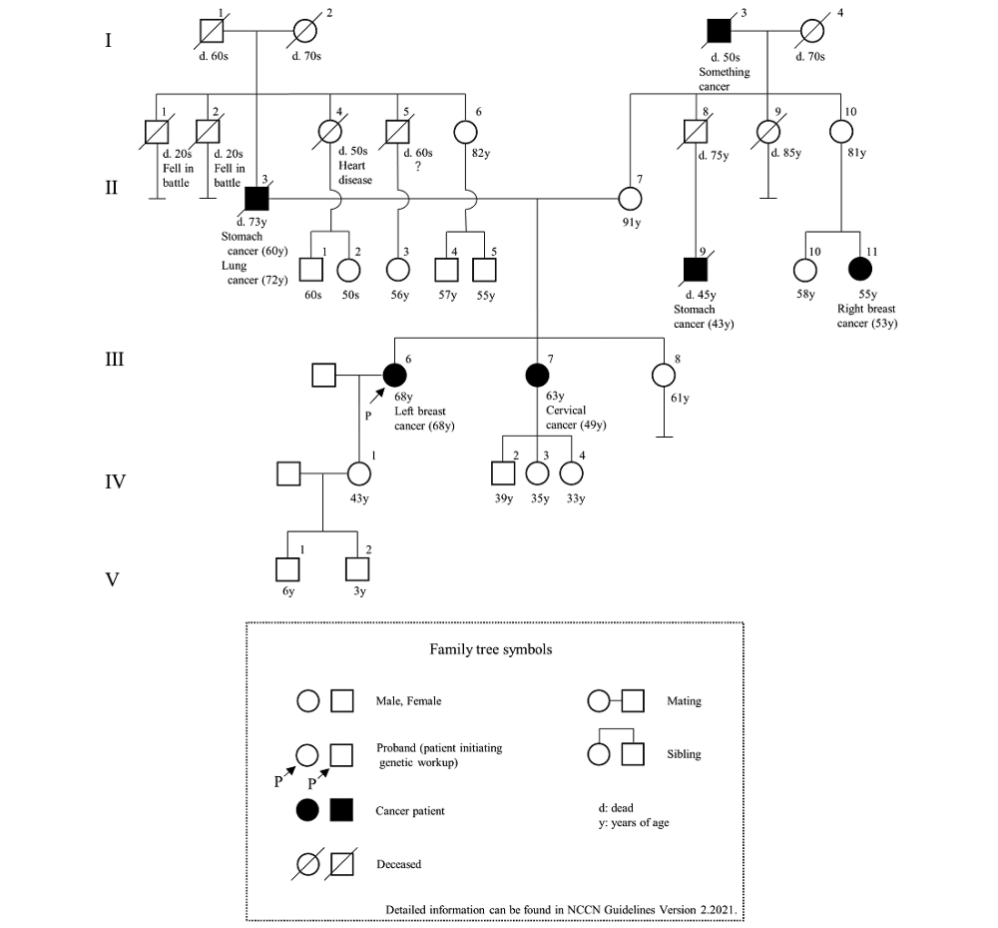
The family tree in scenario 3.

System development and evaluation were conducted by a doctor specializing in genetic medicine (author HN) and 2 CGCs (authors AS and EH). We prepared 3 scenarios with 3 pedigrees (Figures 1-3) and created a conversation with the chatbot along the lines of these scenarios. Then, we evaluated chatbot feasibility, the required time, the medical accuracy of the conversation and the family history, and the final result.

To test the system, 3 experts used this system based on their family histories and evaluated the accuracy of the family histories and the final results.

## Results

**Figure 4:**
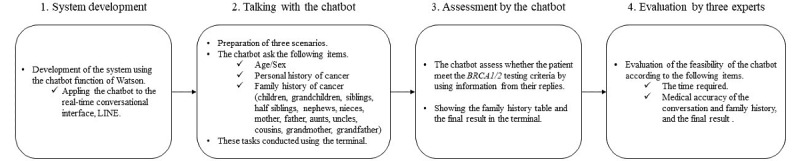
The algorithm of the study.

**Figure 5:**
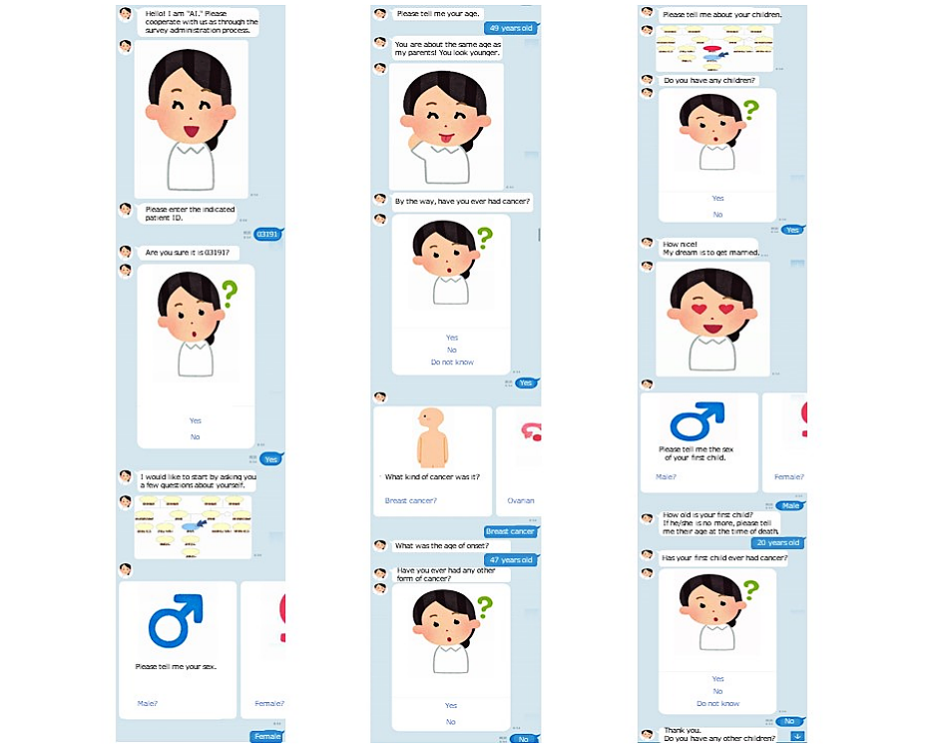
The interface of the chatbot. The LINE interface was used. The conversation in Japanese was translated into English.

**Figure 6:**
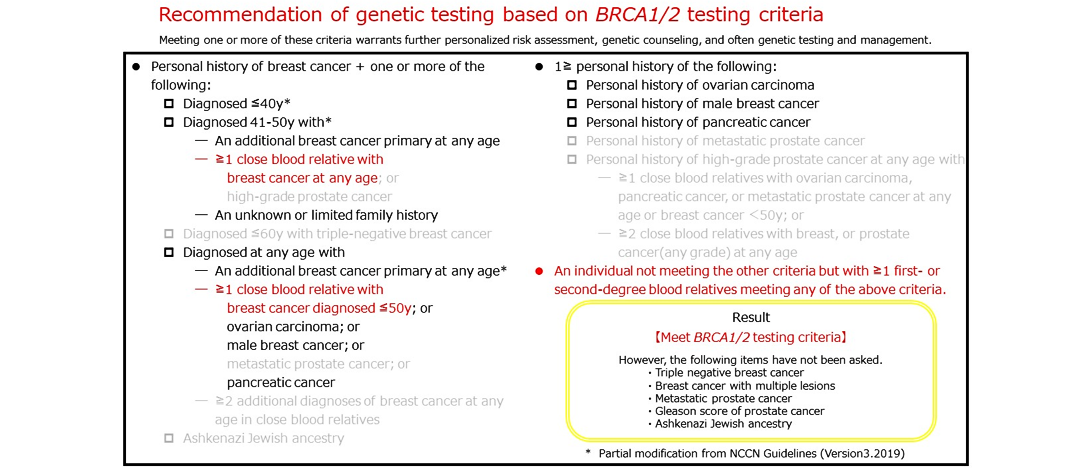
Snapshot of the results of a preliminary screening by the chatbot for scenario 1, according to the criteria of the National Comprehensive Cancer Network (NCCN). We have partially modified the criteria according to our clinical practice at the Kanagawa Cancer Center. The results were originally presented in Japanese and translated into English. Black font: unmet items; red font: met items; gray font: items that had not been asked by this system; yellow square: the final result; “close blood relative:” includes first-, second-, and third-degree relatives.

**Figure 7:**
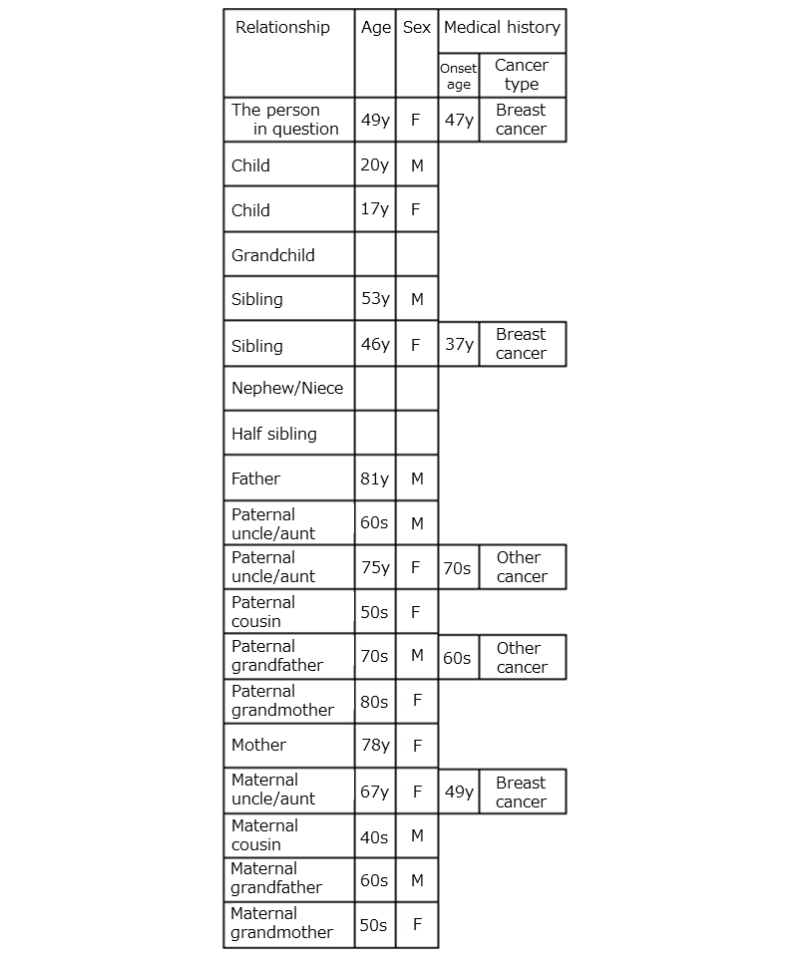
Snapshot of the family history obtained as a result of the preliminary screening by the chatbot for scenario 1. The results were originally presented in Japanese and translated into English. The screen of the terminal presents one of the results. F: female; M: male: y: years of age.

We developed a system that applied the chatbot to the LINE interface (Figure 4). We then created a conversation with the chatbot according to the 3 devised scenarios. The interface is shown in Figure 5, and the contents of the conversation are shown in Multimedia Appendices 1-3. Figures 6 and 7 show examples of the results obtained during a conversation with the chatbot. Scenarios 1 and 2 met the criteria, whereas scenario 3 did not. This result agreed with the assessments of the 3 experts. Specifically, the comparisons of the family histories detected by the chatbot with the actual scenarios indicated that each result was consistent with each scenario. The times required for the conversations were 7 minutes for scenario 1, 15 minutes for scenario 2, and 16 minutes for scenario 3. The 3 experts retrospectively reviewed the conversations with the chatbot and noted no errors from the perspective of genetic medicine.

The 3 experts also confirmed the accuracy of the presented family histories and evaluated the accuracy of the presented final test results using the chatbot system.

## Discussion

### Principal Findings

This study demonstrated that the chatbot system could be applied to preliminary screening for HBOC in a genetic medicine setting. Our developed system allowed us to achieve the following points required for clinical application: First, our system automatically asked for information items about family history that addressed most of the NCCN guidelines. Thus, the preliminary screening can be performed by a chatbot rather than by a CGC. It cannot completely substitute for a CGC; however, using a chatbot for simpler tasks such as a primary screening would allow a CGC to focus on more complicated clinical practices that require more effort. Second, it was possible to ensure medical accuracy; although the development of the system received technical support from information technology companies, the design was created by health care professionals. Accordingly, health care professionals can also change and modify the contents of the conversations regarding the clinical application and continue developing the system while ensuring medical accuracy. Third, the number of correspondences could theoretically be increased to an infinite degree, and therefore, the scale could also be increased. Consequently, the numbers of identified high-risk patients and hospitals performing preliminary screenings could be increased. This offers great promise for the field of genetic medicine.

In this study, we communicated with the chatbot by description-type responses and selection-type responses. This approach was adopted to obtain regular information from existing chatbots [14,15]. As a result, the response time could be reduced relative to the time required to provide only descriptive responses. Furthermore, although selection-type responses are frequently used in chatbots that are commercialized for customer services, our preliminary screening scenario was different. Here, the patient did not voluntarily access the chatbot for the purpose of inquiry but was asked to engage with the system by health care professionals. Therefore, it was necessary to devise an order of questions that would enable an easy recall of the family history and develop a persona that would enable the patient to complete the answers without getting tired. However, previous studies have not emphasized the application of the persona setting in chatbots in the medical field [10-12].

When collecting a family history using our newly developed chatbot, we assumed that AI and the patient would discuss family. Therefore, we created detailed family information for AI and included this in her persona. To improve the response rate, the development of more useful and effective personae is warranted in future studies. It would also be useful to personalize the chat, which is based on the persona of AI, by selecting from among several personae according to the patient’s age. Moreover, chatting based on the persona of AI may or may not be considered excessive, depending on the patient’s background.

### Limitations

Several issues should be addressed to ensure the practical application of this system in terms of efficiency and convenience. First, the system must be easy to operate by the patient. A user-friendly interface is desirable. Therefore, we developed a chatbot system using the LINE interface. As noted, LINE is the most popular SNS in Japan, and we assumed that it would be familiar to the patients [13]. However, older adults who are not used to smartphones may need a friendlier interface or human assistance. In Japan, the personal ownership rate and use of smartphones have been increasing since 2010. Remarkably, the associated generational gap is large, as only 18.8% and 6.1% of those in their 70s and 80s have reported owning such phones, respectively, compared to more than 90% of those in their 20s and 30s [16]. Therefore, the development of a system that considers both age and information technology literacy would be required. Although the supporting personnel would not necessarily need to be CGCs, sufficient personnel would be required to support multiple patients in parallel. Moreover, both elderly and visually impaired patients may find it difficult to operate a smartphone without voice assistance. In this case, it may be difficult to determine the significance of using the AI screening system.

Second, the patient must complete all queries during the screening process. In addition to the persona setting, a device that does not bore the patient is required. A change in the depth of the information heard during preliminary screening would also be necessary. We identified high-risk patients in our institution by interviewing all patients with breast cancer who visited the outpatient department (preliminary screening). As there is a limited amount of time before the medical examination, the information reported by the patient is often limited to relatives who have had cancer rather than the family structure (grandparents, parents, uncles and aunts, cousins, siblings, nephews and nieces, and children) and medical histories. Although our new system allows us to listen to each person's medical history after determining the family structure, it may be useful to adjust the system further depending on the purpose (eg, focusing only on family members suffering from cancer).

Third, we must consider how to handle personal information. We developed a system that is interacted with using a terminal (ie, a smartphone or tablet). However, the secure retention of data requires further exploration. The linking of the obtained results with electronic medical records would be an appropriate handling method.

Finally, clinical trials using this system are required; here, the device would be returned to the health care professionals once the patient has finished the conversation. In this study, the experts responded to the items based on the created scenarios. Evaluation by 3 predefined scenarios is limited system validation. Thus, we have conducted a study with a small number of patients. We would expect that the time required to input answers for actual patients would be longer because they would also need to recall the family history. In addition, human error (such as incorrectly entering a patient ID or confusing the appropriate terminal with another patient’s terminal) may occur when patients being screened are also simultaneously treated at the outpatient department. Measures to prevent such errors may also be required. Therefore, it is first necessary to conduct further studies to solve any problem with this system on a small number of people before further investigation is engaged.

### Conclusions

We demonstrated that the chatbot system could be applied to preliminary screening for HBOC in a genetic medicine setting. Our system could automatically ask for family history items that covered most of the NCCN guidelines without requiring an actual person and remained automated up to the screening result. Health care professionals determined the system design; thus, it was possible to ensure medical accuracy. Theoretically, the number of correspondences and interactions could be increased to an infinite degree, and therefore, the scale could also be increased. The system may also apply to other diseases for which the screening criteria are based on family history. For future clinical applications, it will be necessary to conduct clinical research and to further improve the efficiency and convenience of the system.

## Appendix

Multimedia Appendix 1 Summary and chat contents of scenario 1.

Multimedia Appendix 2 Summary and chat contents of scenario 2.

Multimedia Appendix 3 Summary and chat contents of scenario 3.

## Abbreviations

AI: augmented intelligence
BRCA1/2: BRCA1 or BRCA2
CGC: certified genetic counselor
HBOC: hereditary breast and ovarian cancer
NCCN: National Comprehensive Cancer Network
SNS: social network service
