# Supplementary material for: Preliminary Screening for Hereditary Breast and Ovarian Cancer Using a Chatbot Augmented Intelligence Genetic Counselor: Development and Feasibility Study
Source: JMIR Form Res. 2021 Feb 5;5(2):e25184. doi: 10.2196/25184 (PMC7895643; doi:10.2196/25184)
Supplement: Multimedia Appendix 2 [file formative_v5i2e25184_app2.docx]

| **Supplementary table 2: Summary and chat contents of scenario 2.** | |
| --- | --- |
| AI | Hello! I am "AI." Please cooperate with us as through the survey administration process. |
| AI | Please enter the indicated patient ID. |
| Human | 03192 |
| AI | Are you sure it is 03192? {Yes or No} |
| Human | Yes |
| AI | I would like to start by asking you a few questions about yourself. |
| AI | Please tell me your sex. {Male or Female} |
| Human | Female |
| AI | Please tell me your age. |
| Human | 49 years old |
| AI | You are about the same age as my parents! You look younger. |
| AI | By the way, have you ever had cancer? {Yes or No or Do not know} |
| Human | Yes |
| AI | What kind of cancer was it? {Breast cancer or Ovarian cancer or Prostate cancer or Pancreatic cancer or Other cancer or Do not know} |
| Human | Breast cancer |
| AI | What was the age of onset? |
| Human | 49 years old |
| AI | Have you ever had any other form of cancer? {Yes or No} |
| Human | No |
| AI | Please tell me about your children. |
| AI | Do you have any children? {Yes or No} |
| Human | Yes |
| AI | How nice! My dream is to get married. |
| AI | Please tell me the sex of your first child. {Male or Female} |
| Human | Female |
| AI | How old is your first child? If he/she is no more, please tell me their age at the time of death. |
| Human | 20 years old |
| AI | Has your first child ever had cancer? {Yes or No or Do not know} |
| Human | No |
| AI | Thank you. Do you have any other children? {Yes or No} |
| Human | Yes |
| AI | Having many children must make you happy! The birth rate is low in Japan. It is less than 1.5! |
| AI | Please tell me the sex of your second child. {Male or Female} |
| Human | Male |
| AI | How old is your second child? If he/she is no more, please tell me their age at the time of death. |
| Human | 17 years old |
| AI | Has your second child ever had cancer? {Yes or No or Do not know} |
| Human | No |
| AI | Thank you. Do you have any other children? {Yes or No} |
| Human | No |
| AI | Please tell me about your grandchildren. |
| AI | Do you have any grandchildren? {Yes or No} |
| Human | No |
| AI | Please tell me about your siblings. |
| AI | Do you have any siblings? {Yes or No} |
| Human | Yes |
| AI | Please tell me the sex of your first sibling. {Male or Female} |
| Human | Female |
| AI | How old is your first sibling? If he/she is no more, please tell me their age at the time of death. |
| Human | 54 years old |
| AI | That reminds me, I have a younger brother in university. He does not study hard. So, I worry about whether he will succeed in graduating from university and in getting a job. |
| AI | So, has your first sibling ever had cancer? {Yes or No or Do not know} |
| Human | No |
| AI | Thank you. Do you have any other siblings? {Yes or No} |
| Human | Yes |
| AI | Please tell me the sex of your second sibling. {Male or Female} |
| Human | Male |
| AI | How old is your second sibling? If he/she is no more, please tell me their age at the time of death. |
| Human | 45 years old |
| AI | I have an older sister. We used to quarrel sometimes when we were children. She is a mother now. |
| AI | So, has your second sibling ever had cancer? {Yes or No or Do not know} |
| Human | No |
| AI | Thank you. Do you have any other siblings? {Yes or No} |
| Human | No |
| AI | Please tell me about your nephews and nieces. |
| AI | Do you have any nephews or nieces? |
| AI | I mean the children of blood-related siblings. {Yes or No} |
| Human | Yes |
| AI | I saw my niece over the New Year holidays. She goes to kindergarten. She told me, "My classmate told me that he loves me." |
| AI | Please tell me the sex of your first nephew or niece. {Male or Female} |
| Human | Male |
| AI | How old is your first nephew or niece? If he/she is no more, please tell me their age at the time of death. |
| Human | 25 years old |
| AI | Has your nephew or niece ever had cancer? {Yes or No or Do not know} |
| Human | No |
| AI | Thank you. Do you have any other nephews or nieces? {Yes or No} |
| Human | Yes |
| AI | My niece told me that she does not like him because he is not popular in class. |
| AI | Please tell me the sex of your second nephew or niece. {Male or Female} |
| Human | Male |
| AI | How old is your second nephew or niece? If he/she is no more, please tell me their age at the time of death. |
| Human | 23 years old |
| AI | Has your nephew or niece ever had cancer? {Yes or No or Do not know} |
| Human | No |
| AI | Thank you. Do you have any other nephews or nieces? {Yes or No} |
| Human | Yes |
| AI | I think she must have been glad! I would be glad if anbody told me that they loved me. I enjoyed talking with the five-year-old girl. |
| AI | Please tell me the sex of your third nephew or niece. {Male or Female} |
| Human | Female |
| AI | How old is your third nephew or niece? If he/she is no more, please tell me their age at the time of death. |
| Human | 10 years old |
| AI | Has your nephew or niece ever had cancer? {Yes or No or Do not know} |
| Human | No |
| AI | Thank you. Do you have any other nephews or nieces? {Yes or No} |
| Human | Yes |
| AI | Please tell me the sex of your fourth nephew or niece. {Male or Female} |
| Human | Female |
| AI | How old is your fourth nephew or niece? If he/she is no more, please tell me their age at the time of death. |
| Human | 7 years old |
| AI | Has your nephew or niece ever had cancer? {Yes or No or Do not know} |
| Human | No |
| AI | Thank you. Do you have any other nephews or nieces? {Yes or No} |
| Human | No |
| AI | If you have any half-siblings, please tell me about them. By "half-sibling," I mean a "child who was born after one's mother was remarried." |
| AI | Do you have any half siblings? {Yes or No} |
| Human | No |
| AI | Please tell me about your father. |
| AI | My father is very strict. My curfew is 10:00 pm. Very strict! Now, he is nervous about his health condition. His health-check exam did not come back clean. |
| AI | How old is your father? If he/she is no more, please tell me their age at the time of death. |
| Human | 82 years old |
| AI | Has your father ever had cancer? {Yes or No or Do not know} |
| Human | Yes |
| AI | What kind of cancer was it? {Breast cancer or Ovarian cancer or Prostate cancer or Pancreatic cancer or Other cancer or Do not know} |
| Human | Other cancer |
| AI | What was the age of onset? |
| Human | 50s |
| AI | Has he/she ever had any other form of cancer? {Yes or No} |
| Human | No |
| AI | Please tell me about your mother. |
| AI | My mother has a laid-back personality. My parents' personalities are quite different. I do not known why they are attracted to each other. She is a nurse. She was a good chief nurse in the hospital that she worked for. |
| AI | How old is your mother? If he/she is no more, please tell me their age at the time of death. |
| Human | 79 years old |
| AI | Has your mother ever had cancer? {Yes or No or Do not know} |
| Human | No |
| AI | Do you have any uncles or aunts on your father's side? {Yes or No} |
| Human | Yes |
| AI | My uncle has begun to engage in a new hobby, that is, making smoked food in his garden. His smoked duck is very good. |
| AI | Please tell me the sex of your first uncle or aunt on your father's side. {Male or Female} |
| Human | Female |
| AI | How old is your first uncle or aunt on your father's side? If he/she is no more, please tell me their age at the time of death. |
| Human | 50s |
| AI | Has your first uncle or aunt on your father's side ever had cancer? {Yes or No or Do not know} |
| Human | Yes |
| AI | What kind of cancer was it? {Breast cancer or Ovarian cancer or Prostate cancer or Pancreatic cancer or Other cancer or Do not know} |
| Human | Breast cancer |
| AI | What was the age of onset? |
| Human | 47 years old |
| AI | Has he/she ever had any other form of cancer? {Yes or No} |
| Human | No |
| AI | Do you have any other uncles or aunts on your father's side? {Yes or No} |
| Human | Yes |
| AI | My uncle sometimes showed me his original magic tricks when I was a child. It was great fun. |
| AI | Please tell me the sex of your second uncle or aunt on your father's side. {Male or Female} |
| Human | Male |
| AI | How old is your second uncle or aunt on your father's side? If he/she is no more, please tell me their age at the time of death. |
| Human | 79 years old |
| AI | Has your second uncle or aunt on your father's side ever had cancer? {Yes or No or Do not know} |
| Human | No |
| AI | Thank you. Do you have any other uncles or aunts on your father's side? {Yes or No} |
| Human | Yes |
| AI | Please tell me the sex of your third uncle or aunt on your father's side. {Male or Female} |
| Human | Female |
| AI | How old is your third uncle or aunt on your father's side? If he/she is no more, please tell me their age at the time of death. |
| Human | 75 years old |
| AI | Has your third uncle or aunt on your father's side ever had cancer? {Yes or No or Do not know} |
| Human | No |
| AI | Thank you. Do you have any other uncles or aunts on your father's side? {Yes or No} |
| Human | Yes |
| AI | Please tell me the sex of your fourth uncle or aunt on your father's side. {Male or Female} |
| Human | Male |
| AI | How old is your fourth uncle or aunt on your father's side? If he/she is no more, please tell me their age at the time of death. |
| Human | 72 years old |
| AI | Has your fourth uncle or aunt on your father's side ever had cancer? {Yes or No or Do not know} |
| Human | Yes |
| AI | What kind of cancer was it? {Breast cancer or Ovarian cancer or Prostate cancer or Pancreatic cancer or Other cancer or Do not know} |
| Human | Prostate cancer |
| AI | What was the age of onset? |
| Human | 50s |
| AI | Has he/she ever had any other form of cancer? {Yes or No} |
| Human | No |
| AI | Do you have any other uncles or aunts on your father's side? {Yes or No} |
| Human | No |
| AI | Do you have any cousins on your father's side? {Yes or No} |
| Human | Yes |
| AI | Having cousins is a good thing. I often played with my cousins when we were children. |
| AI | Please tell me the sex of your first cousin on your father's side. {Male or Female} |
| Human | Male |
| AI | How old is your first cousin on your father's side? If he/she is no more, please tell me their age at the time of death. |
| Human | 57 years old |
| AI | Has your first cousin on your father's side ever had cancer? {Yes or No or Do not know} |
| Human | No |
| AI | Thank you. Do you have any other cousins on your father's side? {Yes or No} |
| Human | Yes |
| AI | Please tell me the sex of your second cousin on your father's side. {Male or Female} |
| Human | Female |
| AI | How old is your second cousin on your father's side? If he/she is no more, please tell me their age at the time of death. |
| Human | 55 years old |
| AI | Has your second cousin on your father's side ever had cancer? {Yes or No or Do not know} |
| Human | Yes |
| AI | What kind of cancer was it? {Breast cancer or Ovarian cancer or Prostate cancer or Pancreatic cancer or Other cancer or Do not know} |
| Human | Pancreatic cancer |
| AI | What was the age of onset? |
| Human | 54 years old |
| AI | Has he/she ever had any other form of cancer? {Yes or No} |
| Human | No |
| AI | Do you have any other cousins on your father's side? {Yes or No} |
| Human | Yes |
| AI | You have many cousins! I also have many cousins. |
| AI | Please tell me the sex of your third cousin on your father's side. {Male or Female} |
| Human | Male |
| AI | How old is your third cousin on your father's side? If he/she is no more, please tell me their age at the time of death. |
| Human | 47 years old |
| AI | Has your third cousin on your father's side ever had cancer? {Yes or No or Do not know} |
| Human | No |
| AI | Thank you. Do you have any other cousins on your father's side? {Yes or No} |
| Human | Yes |
| AI | Please tell me the sex of your fourth cousin on your father's side. {Male or Female} |
| Human | Male |
| AI | How old is your fourth cousin on your father's side? If he/she is no more, please tell me their age at the time of death. |
| Human | 45 years old |
| AI | Has your fourth cousin on your father's side ever had cancer? {Yes or No or Do not know} |
| Human | No |
| AI | Thank you. Do you have any other cousins on your father's side? {Yes or No} |
| Human | Yes |
| AI | I have many cousins, too! In our last family dinner, we really enjoyed a lot. We took group photos, too! |
| AI | Please tell me the sex of your fifth cousin on your father's side. {Male or Female} |
| Human | Female |
| AI | How old is your fifth cousin on your father's side? If he/she is no more, please tell me their age at the time of death. |
| Human | 42 years old |
| AI | Has your fifth cousin on your father's side ever had cancer? {Yes or No or Do not know} |
| Human | Yes |
| AI | What kind of cancer was it? {Breast cancer or Ovarian cancer or Prostate cancer or Pancreatic cancer or Other cancer or Do not know} |
| Human | I do not know |
| AI | Identifying the exact diagnosis must have been difficult. What was the age of onset? |
| Human | I do not know |
| AI | Remembering the age of onset can be difficult, too. Has he/she ever had any other form of cancer? {Yes or No} |
| Human | No |
| AI | Do you have any other cousins on your father's side? {Yes or No} |
| Human | Yes |
| AI | Please tell me the sex of your sixth cousin on your father's side. {Male or Female} |
| Human | Female |
| AI | How old is your sixth cousin on your father's side? If he/she is no more, please tell me their age at the time of death. |
| Human | 40s |
| AI | Has your sixth cousin on your father's side ever had cancer? {Yes or No or Do not know} |
| Human | No |
| AI | Thank you. Do you have any other cousins on your father's side? {Yes or No} |
| Human | Yes |
| AI | Please tell me the sex of your seventh cousin on your father's side. {Male or Female} |
| Human | Female |
| AI | How old is your seventh cousin on your father's side? If he/she is no more, please tell me their age at the time of death. |
| Human | 39 years old |
| AI | Has your seventh cousin on your father's side ever had cancer? {Yes or No or Do not know} |
| Human | No |
| AI | Thank you. Do you have any other cousins on your father's side? {Yes or No} |
| Human | No |
| AI | Please tell me about your uncles and aunts on your mother's side. |
| AI | Do you have any uncles or aunts on your mother's side? {Yes or No} |
| Human | Yes |
| AI | Please tell me the sex of your first uncle or aunt on your mother's side. {Male or Female} |
| Human | Male |
| AI | How old is your first uncle or aunt on your mother's side? If he/she is no more, please tell me their age at the time of death. |
| Human | 75 years old |
| AI | Has your first uncle or aunt on your father's side ever had cancer? {Yes or No or Do not know} |
| Human | Yes |
| AI | What kind of cancer was it? {Breast cancer or Ovarian cancer or Prostate cancer or Pancreatic cancer or Other cancer or Do not know} |
| Human | Other cancer |
| AI | What was the age of onset? |
| Human | 60s |
| AI | Has he/she ever had any other form of cancer? {Yes or No} |
| Human | No |
| AI | Do you have any other uncles or aunts on your mother's side? {Yes or No} |
| Human | Yes |
| AI | Please tell me the sex of your first uncle or aunt on your mother's side. {Male or Female} |
| Human | Male |
| AI | How old is the second uncle or aunt on your mother's side? If he/she is no more, please tell me their age at the time of death. |
| Human | 73 years old |
| AI | Has your second uncle or aunt on your mother's side ever had cancer? {Yes or No or Do not know} |
| Human | No |
| AI | Thank you. Do you have any other uncles or aunts on your mother's side? {Yes or No} |
| Human | No |
| AI | Please tell me about your cousins on your mother's side. |
| AI | Do you have any cousins on your mother's side? {Yes or No} |
| Human | Yes |
| AI | Please tell me the sex of your first cousin on your mother's side. {Male or Female} |
| Human | Male |
| AI | How old is your first cousin on your mother's side? If he/she is no more, please tell me their age at the time of death. |
| Human | 47 years old |
| AI | Has your first cousin on your mother's side ever had cancer? {Yes or No or Do not know} |
| Human | No |
| AI | Thank you. Do you have any other cousins on your mother's side? {Yes or No} |
| Human | Yes |
| AI | Please tell me the sex of your second cousin on your mother's side. {Male or Female} |
| Human | Female |
| AI | How old is your second cousin on your mother's side? If he/she is no more, please tell me their age at the time of death. |
| Human | 45 years old |
| AI | Has your second cousin on your mother's side ever had cancer? {Yes or No or Do not know} |
| Human | No |
| AI | Thank you. Do you have any other cousins on your mother's side? {Yes or No} |
| Human | Yes |
| AI | Please tell me the sex of your third cousin on your mother's side. {Male or Female} |
| Human | Male |
| AI | How old is your third cousin on your mother's side? If he/she is no more, please tell me their age at the time of death. |
| Human | 44 years old |
| AI | Has your third cousin on your mother's side ever had cancer? {Yes or No or Do not know} |
| Human | No |
| AI | Thank you. Do you have any other cousins on your mother's side? {Yes or No} |
| Human | Yes |
| AI | Please tell me the sex of your fourth cousin on your mother's side. {Male or Female} |
| Human | Male |
| AI | How old is the fourth cousin on your mother's side? If he/she is no more, please tell me their age at the time of death. |
| Human | 41 years old |
| AI | Has your fourth cousin on your mother's side ever had cancer? {Yes or No or Do not know} |
| Human | No |
| AI | Thank you. Do you have any other cousins on your mother's side? {Yes or No} |
| Human | No |
| AI | Please tell me about your grandfather on your father's side. |
| AI | How old is your grandfather on your father's side? If he is no more, please tell me their age at the time of death. |
| Human | I do not know |
| AI | Just a round about number is okay |
| Human | 40s |
| AI | Has your grandfather on your father's side ever had cancer? {Yes or No or Do not know} |
| Human | I do not know |
| AI | How old is your grandmother on your father's side? If she is no more, please tell me their age at the time of death. |
| Human | About 80 years old |
| AI | Has your grandmother on your father's side ever had cancer? {Yes or No or Do not know} |
| Human | No |
| AI | Thank you. Let us talk about your grandfather on your mother's side. |
| AI | How old is your grandfather on your mother's side? If he is no more, please tell me their age at the time of death. |
| Human | 70s |
| AI | Has your grandfather on your mother's side ever had cancer? {Yes or No or Do not know} |
| Human | Yes |
| AI | What kind of cancer was it? {Breast cancer or Ovarian cancer or Prostate cancer or Pancreatic cancer or Other cancer or Do not know} |
| Human | Other cancer |
| AI | What was the age of onset? |
| Human | 70s |
| AI | Has he/she ever had any other form of cancer? {Yes or No} |
| Human | No |
| AI | Thank you. Finally, let us talk about your grandmother on your mother's side. |
| AI | My grandmother died when I was a little child. The day before she get hospitalized, she took me for a walk around our town. I was glad because she rarely ever took me on a walk. But, she collapsed because of her illness and went to the hospital the next day. She died shortly after her hospitalization. |
| AI | How old is your grandmother on your mother's side? If she is no more, please tell me their age at the time of death. |
| Human | 80s |
| AI | Has your grandmother on your mother's side ever had cancer? {Yes or No or Do not know} |
| Human | No |
| AI | Thank you for your cooperation. The survey is over. |
| AI | Please return the tablet to the window as it is. |
